# Promoter methylated microRNAs: Potential therapeutic targets in gastric cancer (Review)

**DOI:** 10.3892/mmr.2014.2780

**Published:** 2014-10-27

**Authors:** XIAOQIANG GUO, JIAZENG XIA, JIANG YAN

**Affiliations:** Department of General Surgery and Center of Translational Medicine, Wuxi Second Hospital Affiliated to Nanjing Medical University, Wuxi, Jiangsu 214002, P.R. China

**Keywords:** microRNA, DNA methylation, gastric cancer, therapeutic target

## Abstract

Gastric cancer (GC) is the fourth most commonly diagnosed type of cancer worldwide and has the second highest mortality rate of all cancer types. Classical genetics alone does not fully explain how GC occurs; however, epigenetics provides a partial explanation with regard to the cause of cancer. DNA methylation, the best-known type of epigenetic marker, represses the expression of tumor-suppressor genes and is involved in the pathogenesis of various types of human cancer, including GC. Micro (mi)RNAs are critical in the initiation, progression, metastasis and invasion of GC through gene regulation. The dysregulation of miRNAs is widely recognized as a hallmark of cancer. Recently, studies concerning DNA methylation of miRNAs in GC have been frequently reported, and these studies deepen the knowledge of how epigenetic regulation of miRNAs results in GC pathogenesis and indicate novel therapeutic strategies for GC. The present review provides an overview of the reported DNA methylation of miRNAs in GC.

## 1. Introduction

Gastric cancer (GC) is one of the most common types of malignant tumor, with GC morbidity and mortality worldwide ranked fourth and second highest, respectively, out of all types of cancer. It is estimated that ~989,600 individuals were diagnosed with GC and 738,000 GC patients succumbed to the disease worldwide in 2008 (1). Thus, GC seriously threatens human health and is a global cause of mortality. With improvements in clinical diagnosis and treatment, the five-year survival rate of patients with early GC has significantly increased, but the prognosis of patients with advanced GC remains poor. This disappointing prognosis is mainly due to a lack of effective therapeutic measures. Although a large number of studies regarding GC pathogenesis have been conducted, the molecular mechanism of GC remains poorly understood.

Epigenetic alterations have been shown to exert a critical role in gastric tumorigenesis. Various types of epigenetic modifications have been detected, of which DNA methylation was the first to be elucidated and has been the most widely analyzed. DNA methylation of CpG islands (CGIs) is a critical mechanism that results in the ectopic expression of genes, including microRNA (miRNA) genes (2,3). Increasing evidence has shown that a number of important cellular functions, such as proliferation, differentiation and apoptosis, are regulated by miRNAs. Furthermore, dysregulated miRNAs have been confirmed to be associated with the development of numerous types of human cancer (4). In recent years, studies concerning DNA methylation of miRNAs in GC have been frequently reported, and these studies deepen the knowledge of how the epigenetic regulation of miRNAs results in GC pathogenesis, indicating novel therapeutic strategies for GC. The present review provides a brief introduction to DNA methylation and miRNAs, and summarizes the role of promoter-associated methylation of miRNAs in GC.

## 2. DNA methylation

### DNA methylation and methyltransferases

DNA methylation, as the best-known epigenetic modification, occurs when a methyl group (CH_3_) is added to the cytosine-C5 position in the CpG dinucleotide (5). In the human genome, the distribution of CpG dinucleotides (CpGs) is classified into two types: Diffused distribution and local accumulation. Approximately 80% of CpGs that usually remain heavily methylated exhibit a diffused distribution in repetitive DNA sequences, whereas the other CpGs that are always unmethylated in healthy tissues exhibit local accumulation (6,7). A ~1 kb genomic region with a CpG cluster that is usually hypomethylated in healthy cells is known as a CGI (8). These CpG-rich sequences, detected in approximately half of human genes, are predominantly located in the promoter region in which transcription is initiated, but may also be occasionally identified in the first exon or in the intronic regions of the gene (9,10). The methylation of promoter CGIs has been found to exert a critical role in the regulation of gene expression, genomic imprinting, the inactivation of the X-chromosome in females and in tumorigenesis (11).

The addition of methyl groups from S-adenosylmethionine to C5 is catalyzed by DNA methyltransferases (DNMTs). The DNMT family includes the following five members: DNMT1, DNMT2, DNMT3a, DNMT3b and DNMT3L. DNMT1, as the most abundant DNMT, is involved in maintaining the methylation patterns by replicating these patterns during the S phase of mitosis (12), whereas DNMT3a and 3b are involved in *de novo* methylation, which is associated with normal development and disease (13). DNMT1 cooperates with the DNMT3 family to establish and maintain the CGI methylation patterns. However, DNMT2 exerts limited effects in the methylation of CGIs in DNA and DNMT3L is deficient in catalytic activity, although the latter molecule may enhance DNMT3a/3b catalytic activity through direct binding to the catalytic domains (14). Various DNMT inhibitors have been employed in attempts to treat a number of human diseases, including cancer, caused by CGI DNA methylation; 5-aza-2′-deoxycytidine (5-aza-CdR) may be the most commonly utilized. 5-Aza-CdR is a cytidine analog, which may be incorporated into DNA nucleotides and be covalently coupled with DNMTs, resulting in DNMT dysfunction (15). 5-Aza-CdR has been widely employed to reactivate tumor-suppressor genes that have been silenced due to the high expression levels of DNMTs (16).

### DNA methylation and GC

Since the first study was published in 1983 (17), the association between DNA methylation and cancer has been widely investigated. There is increasing evidence that abnormal DNA methylation is a critical mechanism in the pathogenesis of cancer. Aberrant methylation predominantly consists of hypermethylation or hypomethylation. DNA hypomethylation is primarily global, and usually occurs in repetitive DNA sequences, such as the Alu and LINE sequences. However, gene-specific hypomethylation, occurring in certain distinct regions, particularly promoter-associated CGIs, has also been observed. Genome-wide hypomethylation may result in chromosomal instability, reactivation of transposable elements and loss of imprinting (6,18), and gene-specific hypomethylation is correlated with the upregulation of oncogenes (19,20). However, although hypomethylation was first reported earlier than hypermethylation, the hypermethylation of CGIs in promoter regions has received more attention in recent decades. Furthermore, the mechanism of transcription silencing by promoter CGI hypermethylation is more clearly understood than the carcinogenic mechanism of DNA hypomethylation. Methylated CGIs promote chromatin structural stability; the binding of transcription factors to CGIs is inhibited, which results in the silencing of genes (21). The expression of the majority of tumor-suppressor and DNA repair genes is regulated by CGI methylation, and hypermethylation in the promoter region of these genes may result in the inactivation of genes through transcription silencing, which contributes to the formation of cancer. In addition, according to the two-hit hypothesis proposed by Knudson (22), DNA hypermethylation of tumor-suppressor genes acts as the second hit following gene mutation, which is the first. Furthermore, compared with mutations, unusual methylations in the promoter region are more common and may be detected more easily. Studies examining numerous types of cancer, such as gastric and colorectal cancer, have shown that a change in hypomethylation status does not affect the hypermethylation of CGIs in the promoter, which suggests no clear association between genome-wide hypomethylation and regional hypermethylation (23).

Abnormal methylation in the form of both DNA hypomethylation and local hypermethylation has been observed in GC (24,25). In a number of genes, as compared with genome-wide demethylation, more attention has been focused on increased methylation in promoter-associated CGIs in GC. Increasing evidence has indicated that the aberrant DNA methylation of tumor-suppressor genes is involved in development, progression, metastasis and invasion of GC (25). At present, numerous protein-coding tumor suppressor genes have been demonstrated to exhibit abnormal promoter-associated CGI methylation. These genes are mainly associated with various cellular processes, including regulation of the cell cycle, cell differentiation or apoptosis, signal transduction and DNA repair. In addition, in recent years, the expression of miRNAs has also been identified to be affected by DNA methylation, which contributes to tumorigenesis.

## 3. MicroRNA and cancer

Initially, RNA was identified only as a mediator of the transition of information from DNA to proteins; however, increasing evidence has indicated that RNA exerts a key role in various life processes. miRNA is a type of endogenous, single-stranded, non-coding small RNA, 18–22 nucleotides (nts) in length, which is involved in various biological processes and remains highly conserved during evolution. Since the first report in 1993 (26) and its true recognition in the early 2000s, miRNA has been one of the fastest growing research areas in molecular biology. To date, thousands of miRNAs have been identified in animals and plants, as well as viruses. More than 1,000 miRNAs belong to humans, regulating ~30% of human genes (27).

Subsequent to the downregulation of two miRNAs in chronic lymphocytic leukemia, miR-15 and miR-16, first being reported (28), the association between miRNAs and human cancer has been widely investigated. Due to the finding that miRNAs commonly require only partial sequence homology to the 3′-untranslated region (3′-UTR) of target mRNAs, a single miRNA may have numerous mRNA targets and, conversely, a single mRNA may also be targeted by numerous miRNAs (29). Therefore, miRNAs may regulate numerous mRNAs that are closely associated with various types of cancer. In addition, genome-wide studies have indicated that ~50% miRNAs are located at cancer-associated genomic regions or fragile sites of chromosomes (30), further confirming the closely association between miRNAs and cancer.

miRNAs may function as tumor suppressor genes through the regulation of target genes, which subsequently exhibit lower expression levels. The miR-34 family, itself targeted by p53 via a positive feedback mechanism, is universally inactivated in various types of cancer. miR-34 molecules act as tumor suppressors by regulating the expression of the corresponding targets. For instance, one study found that miR-34a caused the suppression of silent information regulator 1 (SIRT1), which may function as an oncogene. Inhibition of SIRT1 may increase p53 activity and in-turn result in upregulation of miR-34a, to further induce SIRT1 silencing (31).

Conversely, certain miRNAs, such as miR-27a, are overexpressed in cancer and function as oncogenes. miR-27a has been found to be overexpressed in ovarian cancer, breast cancer and GC, and acts as an oncogene through the suppression of targets such as ZBTB10, Myt-1 and prohibitin (32–34). Notably, a few miRNAs act as tumor suppressor genes in certain types of cancer and function as oncogenes in other types of cancer. For instance, miR-25 has been shown to be downregulated in colon cancer tissues, as compared with normal mucosal tissues (35), and has demonstrated the ability to inhibit colon cancer cell growth and migration through downregulation of a target gene, Smad7, which is involved in the proliferation and metastasis of colon cancer (35). However, miR-25 has also been reported to be upregulated in esophageal squamous cell carcinoma (ESCC) tissues, and miR-25 overexpression was observed to induce ESCC cell metastasis and invasion via binding to the 3′UTR of epithelial cadherin (36). These studies demonstrate that the function of miRNAs may be tissue-specific.

## 4. Dysregulation of miRNAs in GC

The dysregulation of miRNAs in GC and the subsequent effects have been widely analyzed. Abnormal expression of miRNAs identified in GC has been implicated in the regulation of the cell cycle, apoptosis, invasion and metastasis. For instance, the miR-222-221 oncogenic cluster, associated with the cell cycle, has been reported to be frequently overexpressed in GC (37). miR-222-221 exhibits greater ectopic expression in GC tissues as compared with normal tissues, and downregulates the protein levels of p27 and p57 through binding to target sites; p21, p27 and p57 are all included in the p21 family, and act as cyclin-dependent kinase (CDK) inhibitors, suppressing the progression of cell cycle (37). miR-15b, miR-16, miR-181b and miR-34 have the same downstream target, B-cell lymphoma 2 (BCL-2), which exhibits an antiapoptotic function; overexpression of these miRNAs inhibits the expression of BCL-2 and induces apoptosis. Furthermore, through the negative regulation of BCL-2 expression, miR-15a, miR-16 and miR-181b may contribute to the repression of multidrug resistance associated with the modulation of apoptosis in human GC cell lines (38–40). miR-21 negatively regulates reversion-inducing-cysteine-rich protein with kazal motifs (RECK), a molecule that represses GC metastasis and angiogenesis, and miR-21 is also involved in cancer tissue invasion and lymph node metastasis via reducing the protein levels of phosphatase and tensin homolog (PTEN), a tumor-suppressor gene (41,42). Furthermore, overexpression of miR-21 leads to inhibition of programmed cell death 4 (PDCD4) expression, which results in lymph node metastasis and venous invasion (43).

**Figure 1:**
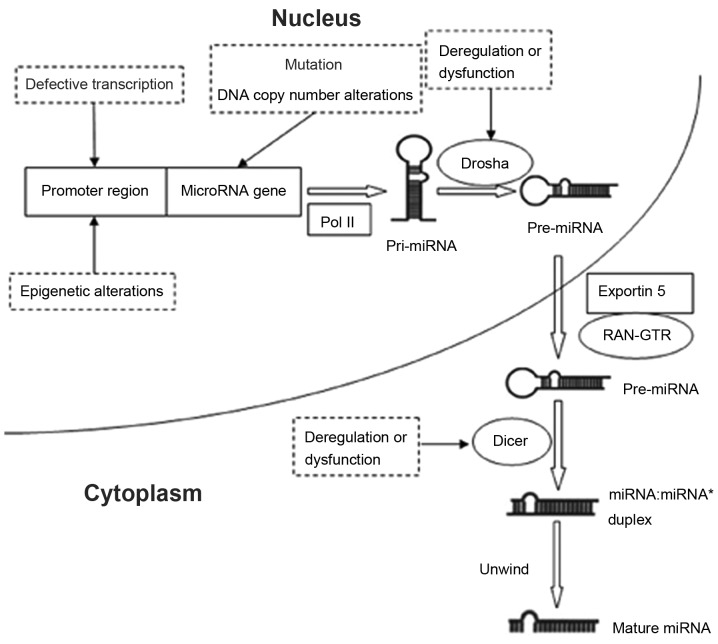
Micro (mi)RNA regulation mechanisms. miRNAs may be regulated by a number of mechanisms, including gene mutation, alteration of DNA copies, defective transcription and dysregulation of miRNA biogenesis components, as well as by epigenetic alteration.

The expression features and the functions of miRNAs have been widely investigated, but the underlying mechanism of miRNA dysregulation is less well-known. However, aberrant miRNA expression has been shown to be mediated by a number of mechanisms, including gene mutation, alteration of the number of DNA copies, defective transcription and dysregulation of miRNA biogenesis components, as well as epigenetic alteration (44) (Fig. 1). Among these mechanisms, DNA methylation may be pivotal in the dysregulation of miRNAs, as methylation mainly represses the expression of tumor-suppressor miRNAs that contain CGIs in promoter regions. In addition, expression of these miRNAs may be restored through demethylation, which indicates that demethylation treatment may serve as a novel therapeutic approach. Thus, DNA methylation of miRNAs requires further investigation in different types of cancer, including GC.

## 5. Aberrant methylation of miRNAs in GC

### miR-124a and miR-34b/miR-34c

Recently, aberrant miRNA methylation in GC has received a great deal of attention. Ando *et al* (45) confirmed that the promoter regions of miR-124a-1, miR-124a-2 and miR-124a-3 were methylated in GC cell lines and samples, which led to the loss of expression of the miRNAs. However, treatment of the cell lines with the 5-aza-CdR demethylation drug resulted in restoration of the miRNAs. This study may have been the first in which DNA methylation was identified as a mechanism of miRNA silencing in GC. Furthermore, the authors found that *H. pylori* infection induced promoter methylation of the miRNAs and increased the risk of GC. In addition, in individuals without *H. pylori* infection, the methylation levels of miR-124a-1, miR-124a-2 and miR-124a-3 were significantly higher in the non-cancerous gastric mucosae of patients with GC than those in the normal mucosae of healthy individuals, which indicates that miR-124a methylation is involved in epigenetic field defects. Therefore, the methylation of members of the miR-124a family may serve a tumor biomarker for the early diagnosis of GC and treatment of *H. pylori* infection may reduce the risk of GC.

In addition to the miR-124a family, miR-34b and miR-34c have also been observed to be silenced by aberrant promoter-associated CGI methylation in the majority of GC cell lines and tissues. Restoration of miR-34b and miR-34c promotes the repression of cell growth, which demonstrates that miR-34b and miR-34c function as tumor-suppressor genes (46). The authors also demonstrated that DNA methylation of miR-34b and miR-34c was associated with *H. pylori* infection in normal individuals, and that the methylation levels of miR-34b and miR-34c in the non-cancerous gastric mucosae of patients with multiple GC were higher than those of patients with single GC. Recently, Suzuki *et al* (47) found that aberrant methylated miR-34b and miR-34c could be an important predictive biomarker of metachronous GC risk. Therefore, the aberrant methylation of miR-34b and miR-34c may be a diagnostic or predictive biomarker, and the re-expression of miR-34b and miR-34c using demethylation drugs may be a novel therapeutic strategy for GC or a useful preventative measure.

### miR-181c

DNA methylation as a mechanism of miRNA silencing in GC has also been identified in miR-181c. Hashimoto *et al* (48) observed that miR-181c expression levels were reduced in 9 of 16 GC samples, as compared with adjacent non-cancerous mucosae, and that miR-181c was upregulated following demethylation treatment of GC cells with 5-aza-CdR. In addition, the authors analyzed the methylation status of miR-181c using bisulfate sequencing and methylation-specific polymerase chain reaction (MSP) analysis, and found that miR-181c was silenced following CGI methylation. These results indicate that reduced miR-181c expression levels are associated with DNA promoter methylation. As with miR-124a and miR-34b/miR-34c, miR-181c methylation signals have also been identified in certain non-cancerous tissues corresponding to GC samples, which implies a defect in the epigenetic field. In order to investigate the effect of miR-181c expression, Hashimoto *et al* (48) transfected two GC cell lines with pre-miR-181c and observed inhibition of cell growth, indicating that miR-181c functions as a tumor-suppressor gene. Through further analysis of the underlying mechanism of action, the authors observed that miR-181c acts via repression of the NOTCH4 and v-Ki-ras2 Kirsten rat sarcoma viral oncogene homolog (KRAS) target genes. NOTCH4 is involved in cell fate determination and KRAS is known as a proto-oncogene that belongs to the RAS family. In conclusion, the study indicated that DNA methylation of miR-181c may be a biomarker of GC and demethylation with epigenetic drugs may be a novel therapeutic approach for GC or other types of cancer. Notably, in this study, miR-181c was only overexpressed in 2 of the 16 GC samples as compared with corresponding non-cancerous tissues. However, Cui *et al* (49) reported that increased expression levels of miR-181c were closely associated with the progression and prognosis of GC. Thus, further studies are required to clarify the exact role of miR-181c in the development, progression and prognosis of GC.

### miR-137

Loss of miR-137 expression has been reported in numerous types of cancer and miR-137 promoter hypermethylation has been observed as an important determinant of miR-137 downregulation in colorectal cancer (50). Therefore, Chen *et al* (51) proposed that miR-137 may be downregulated in GC due to increased methylation of promoter-associated miR-137 CGIs. By use of quantitative polymerase chain reaction and MSP, the authors found that the expression levels of miR-137 were frequently lower in GC tissues than those in the corresponding normal tissues. The authors further demonstrated that miR-137 was expressed at lower levels in methylated GC tissues and that demethylation resulted in the re-expression of miR-137, indicating that miR-137 expression levels are negatively correlated with promoter methylation. In addition, the study demonstrated that transfection of AGC and MKN-45 gastric cancer cell lines with pre-miR-137 inhibited the cell cycle at the G1-S phase and induced apoptosis, which demonstrates that miR-137 may be involved in GC carcinogenesis. As determined by the results of target gene prediction and a luciferase reporter assay, the authors revealed cell division cycle 42 (Cdc42) mRNA to be a direct target of miR-137. Furthermore, cells transfected with pre-miR-137 exhibited reduced expression levels of Cdc42 and Cyclin D1. The authors also found that apoptosis was induced in GC cells transfected with small interfering RNA targeting Cdc42. Therefore, cell cycle arrest may be caused by the inhibition of Cyclin D1 and cell apoptosis may result from the inactivation of Cdc42. The study detected a novel mechanism of GC pathogenesis, which may help in the identification of a novel potential therapeutic target in GC.

### miR-9 and miR-212

In humans, the miR-9 family has three members, miR-9-1, miR-9-2 and miR-9-3, which are located on chromosomes 1, 5 and 15, respectively. Epigenetic repression of miR-9 molecules due to aberrant promoter hypermethylation was first reported in breast cancer (52). Recently, CGI hypermethylation-mediated silencing of all three miR-9 family members was observed in GC; this silencing was reversed in GC cell lines following treatment with 5-aza-CdR (53). Reactivation of miR-9 family members promotes tumor suppressor features, including the repression of cell proliferation and migration. The miR-9 family members may exert tumor-suppressive roles through the inhibition of target genes, such as NF-κB1 and RAB34 (54,55).

As with miR-9, the downregulation of miRNA-212 has been reported to be partly associated with CGI hypermethylation, which is reversed by 5-aza-CdR treatment (2). In addition, miR-212 may function as a tumor suppressor through inhibition of the MYC and MECP2 potential target genes (2). In conclusion, the demethylation of miR-9 and miR-212 may inhibit the progression of GC and these molecules may be novel therapeutic targets.

### miR-148a

Studies have shown that miR-148a is downregulated and DNMT1 is overexpressed in gastric cancer cells. However, the mechanisms underlying the aberrant expression of miR-148a and DNMT1, and their association in gastric cancer remain unknown (56,57). The silencing of miR-148a has been revealed to be associated with promoter hypermethylation in pancreatic cancer (58). Therefore, Zhu *et al* (16) inferred that miR-148a silencing in GC may also be associated with aberrant methylation. This study demonstrated that promoter-associated CGIs were methylated and miR-148a expression levels reduced in the majority of GC samples. As determined by TargetScan miRNA target prediction software, miR-148a may directly target DNMT1, which replicates methylation patterns. Moreover, the results revealed that overexpressed DNMT1 led to miR-148a silencing, whereas restoration of miR-148a resulted in the downregulation of DNMT1. Therefore, there may be a circulating regulation between miR-148a and DNMT1 expression. Furthermore, the authors observed that the reactivation of miR-148a repressed cell growth. In addition, Zheng *et al* (59) identified that overexpression of miR-148 suppressed the metastasis and invasion of GC through repression of a direct target, Rho-associated protein kinase 1 (ROCK1), a potential metastasis promoter. Thus, miR-148a is a potential biomarker of GC prognosis, and restoration of miR-148a may exert an important role in inhibiting the development, metastasis and invasion of GC.

### miR-10b

miR-10b exerts an oncogenic role in numerous types of cancer cell, including breast cancer, malignant glioma, esophageal cancer and hepatocellular carcinoma (HCC) cells, and induces metastasis and invasion by regulation of target genes (60–63). For example, miR-10b is overexpressed in HCC and inhibits the translation of cell adhesion molecule 1 by direct targeting, which promotes HCC metastasis and invasion (60). However, according to results from methylation array and bisulfate pyrosequencing analysis, Kim *et al* (64) reported that miR-10b was downregulated in GC due to promoter region hypermethylation. The authors found that treatment of GC cells with 5-aza-CdR resulted in the re-expression of miR-10b, which further indicated that miR-10b silencing was closely correlated with promoter methylation. Microtubule-associated protein RP/EB family member 1 (MAPRE1), also known as end-binding protein 1, a molecule mapped to chromosome 20q11.2, is upregulated in cancer, and induces cell proliferation and represses apoptosis through the β-catenin/T-cell factor (TCF) signaling pathway, which indicates that MAPRE1 may function as an oncogene (65). Kim *et al* (64) identified that miR-10b silencing in GC cells through methylation led to MAPRE1 overexpression and induced cell proliferation, whereas restoration of miR-10b resulted in inhibition of MAPRE1 and reduced the rate of cell growth and colony formation. In addition, the authors demonstrated using a luciferase reporter assay that miR-10b binds to the 3′UTR of MAPRE1. Therefore, MAPRE1 is a direct and functional target gene of miR-10b, and may repress cell growth through the β-catenin/TCF signaling pathway in GC. In conclusion, DNA methylation of miR-10b may act as a biomarker for estimating the risk of GC and the regulation of miR-10b may have therapeutic potential.

### miR-195 and miR-378

Epigenetic inactivation of miR-195 and miR-378 has recently been reported in GC (66). miRNA silencing in cancer is usually related to abnormal promoter methylation, and thus Deng *et al* (66) hypothesized that the downregulation of miR-195 and miR-378 is associated with promoter-associated methylation. The authors observed that the miR-195 and miR-378 promoters contained CGIs, and that the demethylation treatment of two GC cell lines with 5-aza-CdR resulted in the re-expression of these two miRNAs, which confirmed the hypothesis previously proposed. CDK6 as a direct target of miR-195 in hepatocellular carcinoma cells has been reported and the 3′UTR of vascular endothelial growth factor (VEGF) has been demonstrated to contain a potential binding site for miR-378 (67,68). Therefore, Deng *et al* (66) inferred that CDK6 may be the potential target gene of miR-195 in GC and that VEGF may be a candidate target gene of miR-378. The study results revealed that the expression levels of CDK6 and VEGF were negatively correlated with miR-195 and miR-378 expression levels, respectively, with the latter two molecules inhibiting the expression of the former two. Furthermore, according to the results of analyses conducted using two software packages and luciferase reporter assays, the authors identified CDK6 as a potential direct target of miR-195. CDK6 is an important regulatory molecule of the G1 cell cycle phase and downregulation of CDK6 may result in G1-S phase arrest. Aberrant VEGF expression is associated with angiogenesis, metastasis and repression of apoptosis. The authors also found that miR-195 and miR-378 act as tumor-suppressor genes via the inhibition of cell growth resulting from interruption of the cell cycle. The G0/G1 phase arrest caused by miR-195 may be due to the suppression of CDK6, but the mechanism of G2/M phase arrest caused by miR-378 is not clear. In conclusion, the study indicated that promoter-associated CGI methylation may be a critical factor resulting in the silencing of miR-195 and miR-378, and that the restoration of the two miRNAs may have therapeutic potential in GC.

### Other miRNA molecules

Saito *et al* (69) reported that miR-512-5p became activated following epigenetic treatment of gastric cancer cells with 5-aza-CdR and 4-phenylbutyric acid, which indicates that miR-512-5p may be inactivated by DNA methylation. However, the authors did not further analyze the miR-512-5p methylation status in GC. Promoter-associated CGI methylation has been reported to inhibit the transcriptional activity of miR-196b in GC (20). Unlike the above-described miRNAs, miR-196b functions as an oncogene and exhibits hypomethylation in gene promoter regions in primary GC. A recent study revealed that miR-219-2-3p may act as a tumor suppressor in GC through modulation of extracellular signal-regulated kinase (ERK) 1/2-associated signaling pathways (3). In addition, reduced miR-219-2-3p expression levels may be partly associated with DNA methylation. The lower expression levels of miR-219-2-3p are closely associated with tumor staging, and reactivation may inhibit GC cell proliferation and migration, and induce apoptosis, indicating the potential for miR-219-2-3p as a novel therapeutic target and prognostic indicator. Downregulation of miR-338-3p due to hypermethylation in the promoter region has also been detected in GC (70). miR-338-3p exerts antitumor effects through modulation of the synovial sarcoma X breakpoint protein (SSX2I) oncogene, and the respective promoter methylation status may be a useful diagnostic biomarker of GC. The role of DNA methylation of miRNAs in GC reported in recent years is reviewed in Table I.

## 6. Conclusions and future perspectives

At present, the research regarding the aberrant methylation of miRNAs in GC remains far from complete and studies have predominantly analyzed tumor-suppressor genes. Therefore, more studies are required to identify DNA methylation of novel miRNAs in GC, including oncogenic miRNAs. DNA methylation is a reversible process and demethylation treatment may have great potential in cancer treatment. As DNA methylation is mainly catalyzed by members of the DNMT family, DNMT inhibitors may function as demethylation drugs and act as anticancer agents. Among these demethylation inhibitors, 5-aza-CdR is perhaps the most commonly investigated. However, these demethylation inhibitors not only restore the expression of tumor-suppressor miRNAs but also increase the expression levels of particular oncogentic miRNAs, such as miR-196b. Therefore, as demethylation inhibitors exert a dual function, further studies are required to improve drug targeting. For example, current studies have revealed that miRNA mimics, used in place of miRNAs, effectively alleviated the loss of miRNA expression and may be a potential therapeutic strategy (71).

In conclusion, the methylation of miRNAs is involved in GC pathogenesis; therefore, modification of this process is important in treatment. Thus, more in-depth and extensive studies concerning the methylation of miRNAs are required. With the continuous advancement of miRNA methylation research, great progress in the diagnosis and treatment GC is likely.

## Figures and Tables

**Table I:** Summary of methylated miRNAs in GC.

miRNA	Expression levels in GC tissues	Target genes	References
miR-124a-1-3	Downregulated	-	(39)
miR-34b/c	Downregulated	CDK4, MET, CCNE2	(40,41)
miR-181c	Upregulated/downregulated	NOTCH4, KRAS	(42)
miR-137	Downregulated	Cdc42	(45)
miR-9	Downregulated	NF-κB1, RAB34	(47–49)
miR-212	Downregulated	MYC, MeCP2	(50)
miR-148a	Downregulated	DNMT1, ROCK1	(52,53)
miR-10b	Downregulated	MAPRE1	(58)
miR-195	Downregulated	CDK6	(60)
miR-378	Downregulated	VEGF	(60)
miR-196b	Upregulated	-	(16)
miR-512-5p	Downregulated	MCL1	(63)
miR-219-2-3p	Downregulated	-	(64)
miR-338-3p	Downregulated	SSX2IP	(65)

miRNA, microRNA; GC, gastric cancer; CDK4, cyclin-dependent kinase 4; MET, hepatocyte growth factor receptor; CCNE2, cyclin E2; KRAS, v-Ki-ras2 Kirsten rat sarcoma viral oncogene homolog; Cdc42, cell division cycle 42; DNMT1, DNA methyltransferase 1; ROCK1, Rho-associated protein kinase 1; MAPRE1, microtuble-associated protein RP/EB family member 1; CDK6, cyclin-dependent kinase 6; VEGF, vascular endothelial growth factor; RAB34, member of RAS oncogene family; MeCP2, methyl CpG binding protein 2; SSX2IP, synovial sarcoma X breakpoint protein.

## References

[1] Jemal A, Bray F, Center MM, Ferlay J, Ward E, Forman D (2011). Global cancer statistics. CA Cancer J Clin.

[2] Xu L, Wang F, Xu XF (2011). Down-regulation of miR-212 expression by DNA hypermethylation in human gastric cancer cells. Med Oncol.

[3] Lei H, Zou D, Li Z (2013). MicroRNA-219-2-3p functions as a tumor suppressor in gastric cancer and is regulated by DNA methylation. PLoS One.

[4] Tutar L, Tutar E, Tutar Y (2014). MicroRNAs and Cancer; an Overview. Curr Pharm Biotechnol.

[5] Taby R, Issa JP (2010). Cancer Epigenetics. CA Cancer J Clin.

[6] Dunn BK (2003). Hypomethylation: one side of a larger picture. Ann NY Acad Sci.

[7] Frühwald MC, Plass C (2002). Global and gene-specific methylation patterns in cancer: aspects of tumor biology and clinical potential. Mol Genet Metab.

[8] Robertson KD (2005). DNA methylation and human disease. Nat Rev Genet.

[9] Takai D, Jones PA (2002). Comprehensive analysis of CpG islands in human chromosomes 21 and 22. Proc Natl Acad Sci USA.

[10] Herman JG, Baylin SB (2003). Gene silencing in cancer in association with promoter hypermethylation. N Engl J Med.

[11] Bird A (2002). DNA methylation patterns and epigenetic memory. Genes Dev.

[12] Leonhardt H, Page AW, Weier HU, Bestor TH (1992). A targeting sequence directs DNA methyltransferase to sites of DNA replication in mammalian nuclei. Cell.

[13] Okano M, Bell DW, Haber DA, Li E (1999). DNA methyltransferases Dnmt3a and Dnmt3b are essential for de novo methylation and mammalian development. Cell.

[14] Gowher H, Liebert K, Hermann A, Xu G, Jeltsch A (2005). Mechanism of stimulation of catalytic activity of Dnmt3A and Dnmt3B DNA-(cytosine-C5)-methyltransferases by Dnmt3L. J Biol Chem.

[15] Santi DV, Garrett CE, Barr PJ (1983). On the mechanism of inhibition of DNA-cytosine methyltransferases by cytosine analogs. Cell.

[16] Zhu A, Xia J, Zuo J (2012). MicroRNA-148a is silenced by hypermethylation and interacts with DNA methyltransferase 1 in gastric cancer. Med Oncol.

[17] Feinberg AP, Vogelstein B (1983). Hypomethylation distinguishes genes of some human cancers from their normal counterparts. Nature.

[18] Eden A, Gaudet F, Waghmare A, Jaenisch R (2003). Chromosomal instability and tumors promoted by DNA hypomethylation. Science.

[19] Kwon OH, Park JL, Kim M (2011). Aberrant up-regulation of LAMB3 and LAMC2 by promoter demethylation in gastric cancer. Biochem Biophys Res Commun.

[20] Tsai KW, Hu LY, Wu CW (2010). Epigenetic regulation of miR-196b expression in gastric cancer. Genes Chromosomes Cancer.

[21] Costello JF, Frühwald MC, Smiraglia DJ (2000). Aberrant CpG-island methylation has non-random and tumour-type-specific patterns. Nat Genet.

[22] Knudson AG (2001). Two genetic hits (more or less) to cancer. Nat Rev Cancer.

[23] Ross JP, Rand KN, Molloy PL (2010). Hypomethylation of repeated DNA sequences in cancer. Epigenomics.

[24] Bae JM, Shin SH, Kwon HJ (2012). ALU and LINE-1 hypomethylation in multistep gastric carcinogenesis and their prognostic implications. Int J Cancer.

[25] Zhao C, Bu X (2012). Promoter methylation of tumor-related genes in gastric carcinogenesis. Histol Histopathol.

[26] Lee RC, Feinbaum RL, Ambros V (1993). The C. elegans heterochronic gene lin-4 encodes small RNAs with antisense complementarity to lin-14. Cell.

[27] Lewis BP, Burge CB, Bartel DP (2005). Conserved seed pairing, often flanked by adenosines, indicates that thousands of human genes are microRNA targets. Cell.

[28] Calin GA, Dumitru CD, Shimizu M (2002). Frequent deletions and down-regulation of micro-RNA genes miR15 and miR16 at 13q14 in chronic lymphocytic leukemia. Proc Natl Acad Sci USA.

[29] Lewis BP, Shih IH, Jones-Rhoades MW, Bartel DP, Burge CB (2003). Prediction of mammalian microRNA targets. Cell.

[30] Calin GA, Sevignani C, Dumitru CD (2004). Human microRNA genes are frequently located at fragile sites and genomic regions involved in cancers. Proc Natl Acad Sci USA.

[31] Yamakuchi M, Ferlito M, Lowenstein CJ (2008). miR-34a repression of SIRT1 regulates apoptosis. Proc Natl Acad Sci USA.

[32] Mertens-Talcott SU, Chintharlapalli S, Li X, Safe S (2007). The oncogenic microRNA-27a targets genes that regulate specificity protein transcription factors and the G2-M checkpoint in MDA-MB-231 breast cancer cells. Cancer Res.

[33] Liu T, Tang H, Lang Y, Liu M, Li X (2009). MicroRNA-27a functions as an oncogene in gastric adenocarcinoma by targeting prohibitin. Cancer Lett.

[34] Lai Y, Zhang X, Zhang Z (2013). The microRNA-27a: ZBTB10-specificity protein pathway is involved in follicle stimulating hormone-induced VEGF, Cox2 and survivin expression in ovarian epithelial cancer cells. Int J Oncol.

[35] Li Q, Zou C, Zou C (2013). MicroRNA-25 functions as a potential tumor suppressor in colon cancer by targeting Smad7. Cancer Let.

[36] Xu X, Chen Z, Zhao X (2012). MicroRNA-25 promotes cell migration and invasion in esophageal squamous cell carcinoma. Biochem Biophys Res Commun.

[37] Kim YK, Yu J, Han TS (2009). Functional links between clustered microRNAs: suppression of cell-cycle inhibitors by microRNA clusters in gastric cancer. Nucleic Acids Res.

[38] Ji Q, Hao X, Meng Y (2008). Restoration of tumor suppressor miR-34 inhibits human p53-mutant gastric cancer tumorspheres. BMC Cancer.

[39] Xia L, Zhang D, Du R (2008). miR-15b and miR-16 modulate multidrug resistance by targeting BCL2 in human gastric cancer cells. Int J Cancer.

[40] Zhu W, Shan X, Wang T, Shu Y, Liu P (2010). miR-181b modulates multidrug resistance by targeting BCL2 in human cancer cell lines. Int J Cancer.

[41] Zhang Z, Li Z, Gao C (2008). miR-21 plays a pivotal role in gastric cancer pathogenesis and progression. Lab Invest.

[42] Zhang BG, Li JF, Yu BQ, Zhu ZG, Liu BY, Yan M (2012). microRNA-21 promotes tumor proliferation and invasion in gastric cancer by targeting PTEN. Oncol Rep.

[43] Motoyama K, Inoue H, Mimori K (2010). Clinicopathological and prognostic significance of PDCD4 and microRNA-21 in human gastric cancer. Int J Oncol.

[44] Deng S, Calin GA, Croce CM, Coukos G, Zhang L (2008). Mechanisms of microRNA deregulation in human cancer. Cell Cycle.

[45] Ando T, Yoshida T, Enomoto S (2009). DNA methylation of microRNA genes in gastric mucosae of gastric cancer patients: its possible involvement in the formation of epigenetic field defect. Int J Cancer.

[46] Suzuki H, Yamamoto E, Nojima M (2010). Methylation-associated silencing of microRNA-34b/c in gastric cancer and its involvement in an epigenetic field defect. Carcinogenesis.

[47] Suzuki R, Yamamoto E, Nojima M (2013). Aberrant methylation of microRNA-34b/c is a predictive marker of metachronous gastric cancer risk. J Gastroentero.

[48] Hashimoto Y, Akiyama Y, Otsubo T, Shimada S, Yuasa Y (2010). Involvement of epigenetically silenced microRNA-181c in gastric carcinogenesis. Carcinogenesis.

[49] Cui M, Yue L, Fu Y, Yu W, Hou X, Zhang X (2013). Association of microRNA-181c expression with the progression and prognosis of human gastric carcinoma. Hepatogastroenterology.

[50] Balaguer F, Link A, Lozano JJ (2010). Epigenetic silencing of miR-137 is an early event in colorectal carcinogenesis. Cancer Res.

[51] Chen Q, Chen X, Zhang M, Fan Q, Luo S, Cao X (2011). miR-137 is frequently down-regulated in gastric cancer and is a negative regulator of Cdc42. Dig Dis Sci.

[52] Lehmann U, Hasemeier B, Christgen M (2008). Epigenetic inactivation of microRNA gene hsa-mir-9-1 in human breast cancer. J Pathol.

[53] Tsai KW, Liao YL, Wu CW (2011). Aberrant hypermethylation of miR-9 genes in gastric cancer. Epigenetics.

[54] Wan HY, Guo LM, Liu T, Liu M, Li X, Tang H (2010). Regulation of the transcription factor NF-kappaB1 by microRNA-9 in human gastric adenocarcinoma. Mol Cancer.

[55] Luo H, Zhang H, Zhang Z (2009). Down-regulated miR-9 and miR-433 in human gastric carcinoma. J Exp Clin Cancer Res.

[56] Ueda T, Volinia S, Okumura H (2010). Relation between microRNA expression and progression and prognosis of gastric cancer: a microRNA expression analysis. Lancet Oncol.

[57] Mutze K, Langer R, Schumacher F (2011). DNA methyltransferase 1 as a predictive biomarker and potential therapeutic target for chemotherapy in gastric cancer. Eur J Cancer.

[58] Hanoun N, Delpu Y, Suriawinata AA (2010). The silencing of microRNA 148a production by DNA hypermethylation is an early event in pancreatic carcinogenesis. Clin Chem.

[59] Zheng B, Liang L, Wang C (2011). MicroRNA-148a suppresses tumor cell invasion and metastasis by downregulating ROCK1 in gastric cancer. Clin Cancer Res.

[60] Li QJ, Zhou L, Yang F (2012). MicroRNA-10b promotes migration and invasion through CADM1 in human hepatocellular carcinoma cells. Tumour Biol.

[61] Tian Y, Luo A, Cai Y (2010). MicroRNA-10b promotes migration and invasion through KLF4 in human esophageal cancer cell lines. J Biol Chem.

[62] Sasayama T, Nishihara M, Kondoh T, Hosoda K, Kohmura E (2009). MicroRNA-10b is overexpressed in malignant glioma and associated with tumor invasive factors, uPAR and RhoC. Int J Cancer.

[63] Ma L, Teruya-Feldstein J, Weinberg RA (2007). Tumour invasion and metastasis initiated by microRNA-10b in breast cancer. Nature.

[64] Kim K, Lee HC, Park JL (2011). Epigenetic regulation of microRNA-10b and targeting of oncogenic MAPRE1 in gastric cancer. Epigenetics.

[65] Liu M, Yang S, Wang Y (2009). EB1 acts as an oncogene via activating beta-catenin/TCF pathway to promote cellular growth and inhibit apoptosis. Mol Carcinog.

[66] Deng H, Guo Y, Song H (2013). MicroRNA-195 and microRNA-378 mediate tumor growth suppression by epigenetical regulation in gastric cancer. Gene.

[67] Xu T, Zhu Y, Xiong Y, Ge YY, Yun JP, Zhuang SM (2009). MicroRNA-195 suppresses tumorigenicity and regulates G1/S transition of human hepatocellular carcinoma cells. Hepatology.

[68] Hua Z, Lv Q, Ye W (2006). MiRNA-directed regulation of VEGF and other angiogenic factors under hypoxia. PLoS One.

[69] Saito Y, Suzuki H, Tsugawa H (2009). Chromatin remodeling at Alu repeats by epigenetic treatment activates silenced microRNA-512-5p with downregulation of Mcl-1 in human gastric cancer cells. Oncogene.

[70] Li P, Chen X, Su L (2013). Epigenetic silencing of miR-338-3p contributes to tumorigenicity in gastric cancer by targeting SSX2IP. PLoS One.

[71] Bader AG, Brown D, Winkler M (2010). The promise of microRNA replacement therapy. Cancer Res.

